# Prescription of essential medication during the final hospitalization of patients with heart failure or cancer

**DOI:** 10.1186/s12904-025-01682-w

**Published:** 2025-02-26

**Authors:** Valentina González-Jaramillo, Monika Hagemann, Lukas Hunziker, Maud Maessen, Steffen Eychmüller

**Affiliations:** 1 University Centre for Palliative Care, Inselspital, Bern University Hospital, University of Bern, Bern, Switzerland; 2 Department of Cardiology, Inselspital, Bern University Hospital, University of Bern, Bern, Switzerland; 3 Institute of Social and Preventive Medicine (ISPM), University of Bern, Bern, Switzerland

**Keywords:** Heart failure, Cancer, Palliative care, Palliative care, End of life care

## Abstract

**Background:**

Four medication types—opioids, benzodiazepines, anticholinergics, and antipsychotics—have been proposed as essential for patients in their final days, regardless of their primary diagnosis. These drugs are typically prescribed for individuals with cancer who are under specialized palliative care (PC+). However, it is not known whether their usage is equally common for patients with other chronic and progressive conditions, such as heart failure (HF), or for those who are not under specialized palliative care (PC-).

**Aim:**

To assess the prescription frequency of each of the four medication types during the final hospitalization of patients with HF and compare it with the prescription frequency in patients with cancer (CA), considering both PC + and PC- patients in each disease group.

**Methods:**

A retrospective cohort study included all patients dying in a tertiary hospital between 2016 and 2022. We created three disease groups – “HF,” “CA,” and “HF&CA” – splitting each of them into two groups, depending on whether they received PC. So there were a total of six groups – “HF PC+”, “HF PC-“, “CA PC+”, “CA PC-”, “HF&CA PC+”, and “HF&CA PC-”.

**Results:**

Of the 3,874 patients, 1,921 (59%) had cancer exclusively, 371 (10%) had heart failure exclusively, and 691 (18%) had both. The median length of stay was 9 days (IQR 2–16). Within each diagnosis group, PC + patients had a higher prescription frequency for each medication type than PC- patients. For example, patients who received PC had 12 times the odds of being prescribed opioids than those who did not receive it (*p* < 0.05). Among the six groups, the highest prescription frequency of opioids, benzodiazepines, and anticholinergics was seen in the “HF PC+” group and the lowest in the “HF PC-” group. Antipsychotics were prescribed less frequently in the “HF PC-” and the “CA PC-” groups and were mainly prescribed in the “CA PC+” group.

**Conclusion:**

Across the diagnostic groups, a notable difference in the prescription of the four medication types was observed between PC + and PC- patients. This difference was more pronounced among patients with HF (without cancer).

**Supplementary Information:**

The online version contains supplementary material available at 10.1186/s12904-025-01682-w.

## Background

Every patient has individual and specific needs during the trajectory of a disease, including the dying phase. However, there are general recommendations for medications at the end of life that should be prescribed in this phase, either because the patient would benefit from their use at the time or later [1–3]. These recommendations are intended to cover the most common and most relevant symptoms during the last days of life – pain, breathlessness, anxiety, delirium, agitation, nausea, vomiting, and noisy chest secretions. Some medications can help with more than one symptom. For example, levomepromazine can treat delirium, agitation, nausea, and vomiting, and opioids can help with pain, breathlessness, and anxiety.

The proposed types of medication that should be prescribed in the last days of life are opioids, benzodiazepines, anticholinergics, and antipsychotics [1–3]. These types of medications are not an exclusive domain of palliative care specialists. However, due to the greater expertise of these specialists in symptomatic management during advanced stages of disease and the end of life, it is not known whether these medications are mainly prescribed by palliative care specialists or whether physicians with different trainings also prescribe them similarly during the last days of a persons’ life. Although the use of these four types of medications was initially proposed for people with cancer [4], their use has been extended to people with non-oncologic diseases, such as heart failure [5]. However, to date, it is not known whether there are differences in the prescription of these four types of medication during the last days of life between people with heart failure and people with cancer. This study focuses specifically on heart failure because it is a chronic, progressive condition with a high symptom burden, frequent hospitalizations, and a unique trajectory that necessitates ongoing palliative care. Unlike other cardiac conditions such as atrial fibrillation, which may be managed with rhythm control strategies, or stroke, which often follows an acute or subacute course with rehabilitation-focused care, heart failure patients experience more non-cardiovascular comorbidities [6, 7], persistent symptoms [8], recurrent exacerbations [9], and a variable decline [10], making early integration of palliative care essential for improving quality of life. Therefore, our objectives were (1) to compare the frequency of prescription of the four medication types among patients who died from heart failure and those who died from cancer and (2) to compare the frequency of prescription of these drugs among those who received specialized palliative care during their fatal hospitalization with prescriptions among those who did not receive care by that specialty.

## Methods

We conducted a retrospective cohort study in a tertiary university hospital in Switzerland.

### Data collection and ethics

We collected data from all inpatients who died between January 1, 2016, and December 31, 2022, and who consented to the use of their clinical data. With the support of the hospital’s data science center, we extracted information from administrative records and medical charts. The data extracted included sociodemographic information, such as sex, age, and marital status, and clinical information, such as main hospitalization diagnosis, comorbidities (secondary diagnosis), length of stay (LOS), medication prescribed (whether or not it was administered), date of death, and whether or not the patient received PC, and, if so, at which time during the hospitalization. The main diagnoses and comorbidities were extracted using ICD-10 codes and medications using their generic names. Patients received PC within the PC ward or in another ward through consultation.

### Inclusion and exclusion criteria

The inclusion criteria were adult patients (at least 18 years old) who died in the hospital while hospitalized during the previously defined period. We included only patients who had HF or cancer (CA) as the main or secondary diagnosis. We excluded patients who dissented from further use of their medical data, perinatal deaths and deaths caused by accidents or injuries from external causes, and patients who died in ambulatory care or in the intensive care unit. Patients in the ICU were excluded to focus on medication use patterns in routine inpatient care, as ICU settings involve different goals of care, critically ill populations, and unique medication needs that could confound comparisons between heart failure and cancer patients.

### Data analysis

We created categorical variables (yes/no) for HF or cancer based on ICD-10 codes (supplementary material 1). We also created categorical variables for the following comorbidities based on ICD-10 codes – chronic kidney disease (CKD), chronic obstructive pulmonary disease (COPD), and dementia (supplementary material 1).

Since our goal was to compare patients with HF and patients with cancer, we created two main comparison groups – “HF” for patients with HF and “CA” for patients with cancer. As some patients had both HF and cancer, we created a third group called “HF&CA”. We split each of the three disease groups into two depending on whether they received PC (PC+) or not (PC-), leading to a total of six groups – “HF PC+”, “HF PC-”, “HF&CA PC+”, “HF&CA PC-”, “CA PC+”, and “CA PC-”.

We created four medication categories – opioids, benzodiazepines, anticholinergics, and antipsychotics (supplementary material 2).

### Statistical analysis

We compared the patient characteristics and medications prescribed across the six groups. Data are presented as mean with standard deviation (SD) in the case of normally distributed data and as median with interquartile range (IQR) in the case of non-normally distributed data. We assessed normality using Q–Q plots. Categorical variables are presented as frequencies (%). The differences in the frequency in which each medication type was prescribed across the groups were evaluated using Chi^2^ test. The continuous variables were analyzed using the Mann–Whitney test. All analyses were conducted using STATA 16.

We conducted logistic regression analysis with each of the four medication types as the outcome, PC as the main exposure, and HF, cancer, age, sex, and LOS as covariables. As a sensitivity analysis, we also conducted these regressions analysis, further adjusting for COPD, CKD, and dementia.

## Results

### Baseline characteristics

**Fig. 1:**
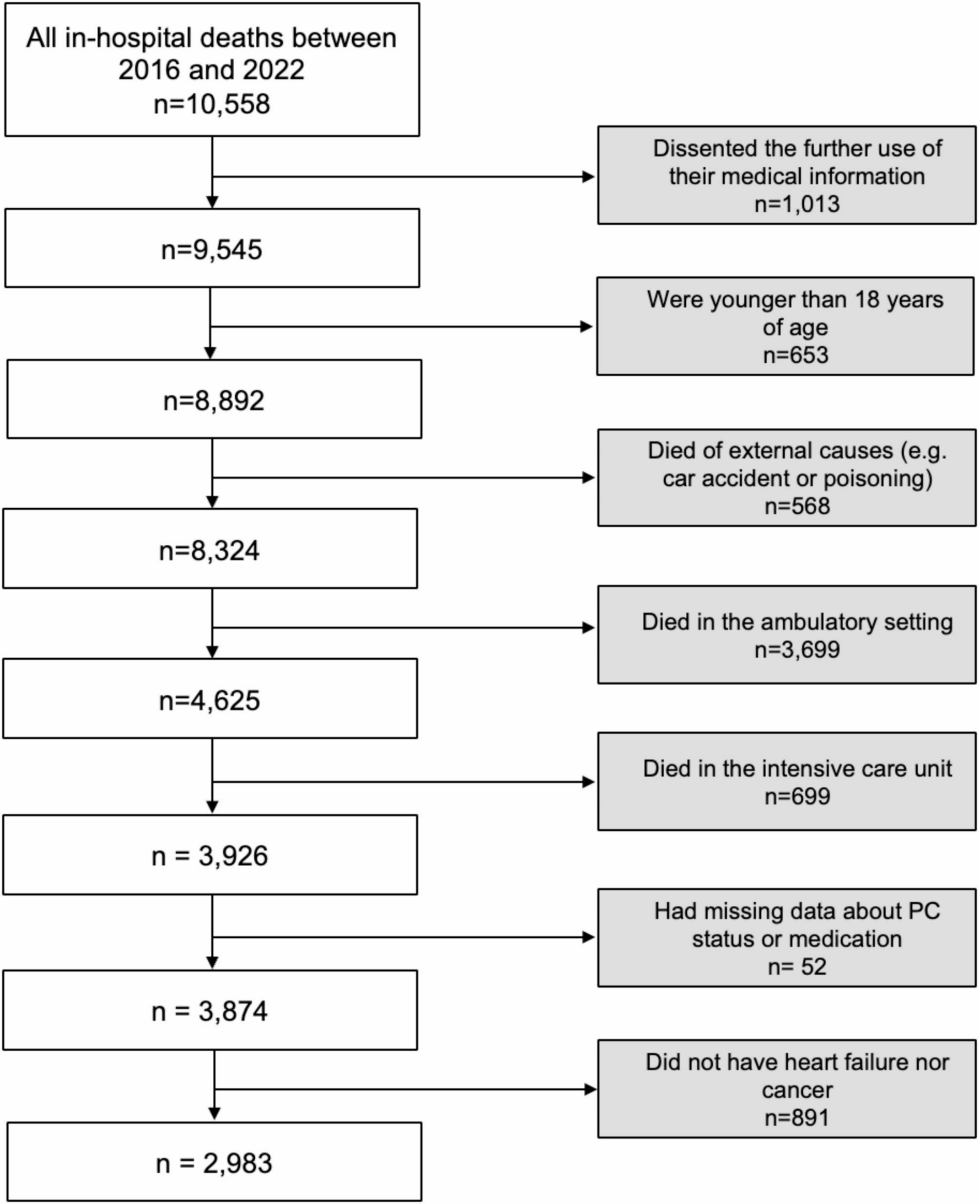
Flowchart of the patients included in the study

Of the 10,558 patients who died in the hospital, 2,983 patients met the inclusion criteria and were included in the study (Fig. 1). Of these, 1,921 (64%) had only cancer, 371 (13%) had only HF, and 691 (23%) had both. The main cancer type was lung cancer (18%), followed by the hematological cancers lymphoma (9%) and leukemia (8%). The majority of the patients were men (*n* = 1841, 62%), and the median age was 74 years (IQR 64–82). The most common comorbidity was CKD, which was present in 30% of the patients (*n* = 898), followed by dementia and COPD, each present in 7% (*n* = 204 and *n* = 217, respectively) of the patients (Table 1).

**Table 1:** Baseline characteristics of the patients included in the study

	Overall				HF				HF & CA				CA				
	2983				371				691				1921				*p*-value
	PC-		PC+		PC-		PC+		PC-		PC+		PC-		PC+		
	2249	75%	734	25%	355	96%	16	4%	584	85%	107	15%	1310	68%	611	32%	< 0.001
Sex																	0.006
Women	876	39%	266	36%	153	43%	4	25%	199	34%	29	27%	524	40%	233	38%	
Men	1373	61%	468	64%	202	57%	12	75%	385	66%	78	73%	786	60%	378	62%	
Age	75	66–84	68	58–75	82	74–89	77	73–86	77	69–84	74	69–79	73	64–81	67	56–75	< 0.001
Comorbidities																	
COPD	179	8%	38	5%	41	12%	2	13%	70	12%	8	7%	68	5%	28	5%	< 0.001
Dementia	182	8%	21	3%	50	14%	3	19%	55	9%	6	6%	77	6%	12	2%	< 0.001
CKD	714	32%	184	25%	133	37%	11	69%	289	49%	58	54%	292	22%	115	19%	< 0.001
LOS (in days)	6	3–13	12	7–21	3	2–7	11	8–17	9	5–16	13	8–20	6	3–13	11	7–21	< 0.001
LOS before PC			3	0–8			6	4–9			6	2–13			3	0–13	< 0.001
LOS after PC			7	3–13			4	3–7			5	3–9			7	3–14	< 0.001

Overall, the median LOS was 8 days (IQR 3–15). The median LOS of patients in the “HF” group was 4 days (IQR 2–7), and patients with heart failure who received PC (4%) received it after a median of 6 days (IQR 4–9). The median LOS of patients in the “CA” group was 8 days (IQR 4–16), and patients with cancer who received PC (*n* = 611, 32%) received it after a median of 3 days (IQR 0–13) (Table 1).

The 4% (*n* = 16) of patients with HF who received PC (patients in the “HF PC+” group) had more frequent instances of COPD, dementia, and CKD and a higher proportion of men compared to patients in the other five groups. While almost half of the “HF” patients were women, a quarter of the “HF PC+” patients were women. The median LOS of “HF PC+” was four times the LOS of the “HF PC-“ patients.

### Medications across disease groups

Among the three diseases groups (“HF”, “HF&CA”, and “CA”), the “HF” group had the lowest proportion of patients that were prescribed each of the four types of medications and the “CA” group the highest except for antipsychotics that were prescribed nearly in the same proportion between “CA” and “HF&CA” patients (Table 2).

**Table 2:** Proportion of patients receiving each medication type across disease groups

	HF (*n* = 371)	HF & CA (*n* = 691)	CA (*n* = 1,921)	*p*-value
Opioids	56%	72%	79%	< 0.001
Benzodiazepines	26%	41%	52%	< 0.001
Anticholinergics	14%	19%	29%	< 0.001
Antipsychotics	17%	25%	24%	< 0.001

### Medications across disease groups by specialized palliative care status

Within each diagnosis group, PC + patients had a higher prescription proportion for all medication types than PC- patients.

Opioids were the most prescribed type of medication, having been prescribed in at least 95% of the patients receiving PC for the three diagnosis groups. Among those who did not receive PC, opioids were prescribed in half of the “HF” patients (*n* = 192, 54%) and in around two-thirds of the “HF&CA” or “CA” patients (*n* = 470, 68% and *n* = 1364, 71%, respectively) (Table 3). Regression analysis showed that the factor that most increased the odds of being prescribed opioids was receiving PC: patients who received PC had 12 times the odds of being prescribed with opioids than those who did not receive it (*p* < 0.05), whereas patients with HF had 33% lower odds of being prescribed with opioids(*p* < 0.05) (Table 4).

**Table 3:** Proportion of patients receiving each medication type across disease groups by palliative care status

	HF (*n* = 371)		HF & CA (*n* = 691)		CA (*n* = 1.921)		
	PC- (*n* = 355)	PC+ (*n* = 16)	PC- (*n* = 584)	PC+ (*n* = 107)	PC- (*n* = 1.310)	PC+ (*n* = 611)	*p*-value
Opioids	54%	100%	68%	95%	71%	97%	< 0.001
Benzodiazepines	23%	94%	36%	69%	38%	82%	< 0.001
Anticholinergics	12%	56%	15%	40%	19%	50%	< 0.001
Antipsychotics	17%	25%	23%	37%	17%	39%	< 0.001

**Table 4:** Logistic regression models for each of the medication types

Logistic regression models			
**A. Opioids**			
	Odds Ratio	[95% Conf. Interval]	*P* value
Specialized palliative care	12.37	8.05-19.00	< 0.001
Heart failure	0.67	0.54–0.84	< 0.001
Cancer	1.36	1.02–1.81	0.038
Age	1.03	1.02–1.04	< 0.001
Sex	1.07	0.89–1.29	0.496
Length of stay	1.80	1.06–1.09	< 0.001
**B. Benzodiazepines**			
	Odds Ratio	[95% Conf. Interval]	*P* value
Specialized palliative care	5.73	4.65–7.07	< 0.001
Heart failure	0.81	0.66–0.97	0.026
Cancer	1.24	0.92–1.67	0.156
Age	0.99	0.99–0.99	0.006
Sex	0.95	0.81–1.12	0.508
Length of stay	1.04	1.03–1.04	< 0.001
**C. Anticholinergics**			
	Odds Ratio	[95% Conf. Interval]	*P* value
Specialized palliative care	4.07	3.35–4.94	< 0.001
Heart failure	0.67	0.54–0.86	0.001
Cancer	1.04	0.72–1.49	0.851
Age	1.00	0.99–1.01	0.692
Sex	1.06	0.88–1.27	0.559
Length of stay	1.01	1.00-1.02	0.031
**D. Antipsychotics**			
	Odds Ratio	[95% Conf. Interval]	*P* value
Specialized palliative care	2.66	2.18–3.26	< 0.001
Heart failure	1.07	0.86–1.33	0.517
Cancer	1.11	0.79–1.55	0.532
Age	1.02	1.01–1.03	< 0.001
Sex	0.72	0.59–0.87	0.001
Length of stay	1.03	1.03–1.04	< 0.001

Benzodiazepines were mainly prescribed for “HF PC+” patients: 94% (*n* = 16) of them were prescribed with this medication, compared to 82% (*n* = 501) of the “CA PC+” group (Table 3). Patients who received PC had almost six times the odds of being prescribed with benzodiazepines than those who did not receive it (*p* < 0.05) and patients with HF had 20% lower odds of being prescribed with this medication (*p* < 0.05) (Table 4).

Anticholinergics were prescribed in approximately half of the patients (*n* = 358) receiving PC and in less than 20% of the patients (*n* = 380) not receiving PC, regardless of the diagnosis group. Patients who received PC had four times the odds of being prescribed with anticholinergics than those who did not receive it (*p* < 0.05), whereas patients with HF had 33% lower odds of being prescribed with anticholinergics(*p* < 0.05) (Table 4).

Finally, antipsychotics were the less frequently prescribed medications and were mainly and similarly prescribed for “CA PC+” and “HF&CA PC+” patients (*n* = 238, 39% and *n* = 40, 37%, respectively) (Table 3). Patients who received PC had 2.5 times the odds of being prescribed with antipsychotics than those who did not receive it (*p* < 0.05), and having HF or cancer were not predictors of being prescribed with antipsychotics (Table 4).

**Fig. 2:**
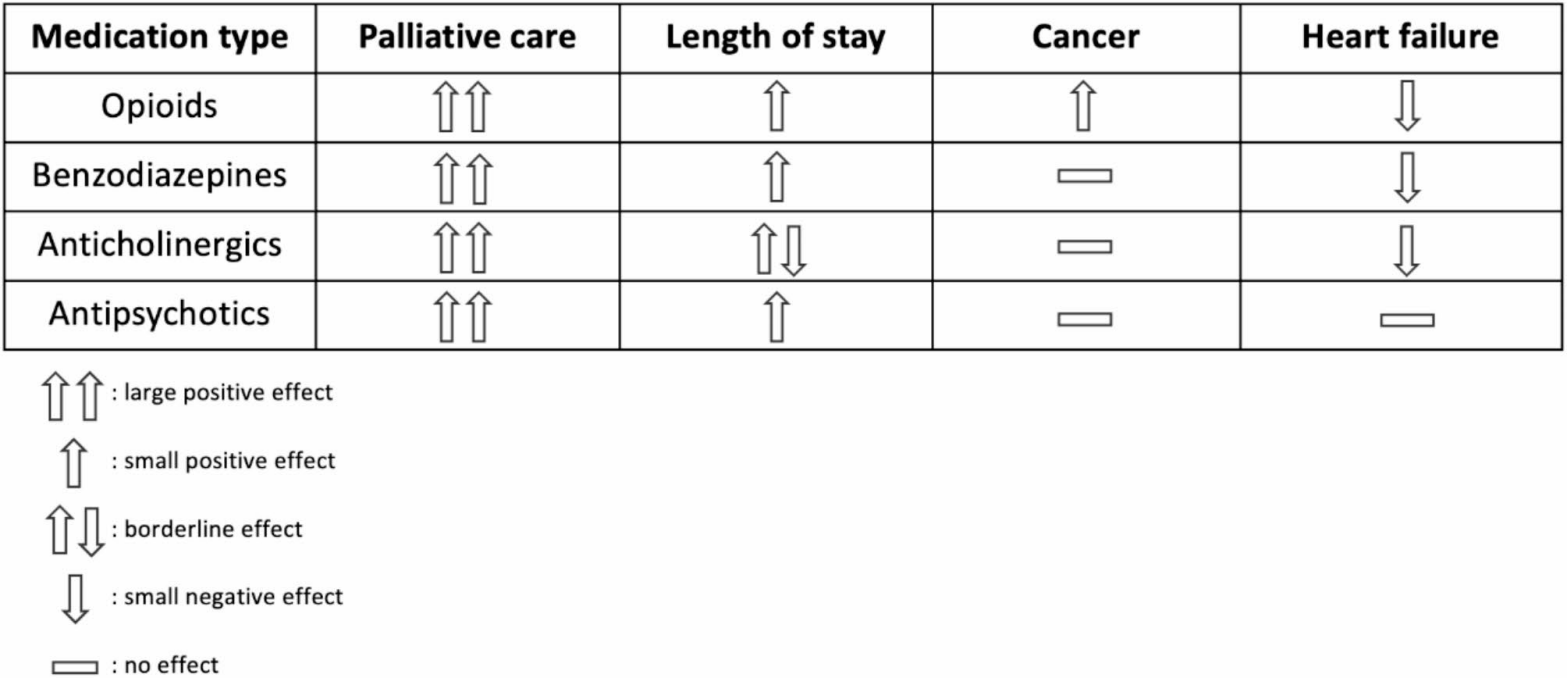
Direction and magnitude of the relation between the four main predictors and each medication type

In the logistic regression analysis, all the variables that were statistically significant in the main model(Table 4), remained significant after further adjustment for CKD, dementia, and COPD. Having received PC and a longer length of stay were statistically significantly associated with increased odds of having received each of the four mediation types. Having HF statistically significantly reduced the odds of receiving opioids, benzodiazepines, and anticholinergics and was not statistically significantly associated with the odds of having received antipsychotics. Finally, having cancer was only statistically significantly associated with higher odds of receiving opioids (Fig. 2).

## Discussion

### Key findings

Patients who received PC were always more frequently prescribed each of the four types of medication than PC- patients, and having received PC was the main predictor for being prescribed each type of medication. Among the six groups, the highest prescription frequency of opioids, benzodiazepines, and anticholinergics was seen in the “HF PC+” group and the lowest in the “HF PC-“group. Antipsychotics were less frequently prescribed between both HF groups and were mainly and equally prescribed in the “HF&CA PC+” and the “CA PC+” groups. Overall, having HF was a negative predictor of being prescribed opioids, benzodiazepines, and anticholinergics, whereas having cancer was a positive predictor of being prescribed opioids.

### Medication prescriptions

Evaluating the frequency of use of certain medications does not necessarily allow us to evaluate the quality of clinical practice. Moreover, not using these medications does not mean that there was a deficiency in clinical practice or that there was a failure in symptom control in patients in whom they were not used, since the medications may not have been indicated or the patients may have refused the use of some of them, such as sedatives. However, it is important to emphasize that in this study, we evaluated the frequency with which the medications were prescribed, not with which they were administered. In this particular case, our results suggest that there was a low anticipatory prescription, which is recommended, of these four types of medications among patients who did not receive PC, especially among those with HF. It is interesting how the prescription frequency of medications was highest among patients with HF who received PC and lowest among patients with HF but without PC. One possible explanation is that HF PC + patients had the most complex symptoms, as they had multiple comorbidities. However, even though this group of patients had the highest overall prevalence of CKD, dementia, and COPD, patients with HF were older than patients with cancer and had more comorbidities, regardless of whether they received PC. This high chance of physical suffering due to age-related frailty and comorbidities is one of the possible reasons patients with HF have comparable or even higher PC needs compared to patients with cancer [11]. The reasons for this large discrepancy in medication use between HF and cancer remain to be explained in future studies.

### Low and late referral to specialized palliative care

In our study, very few patients with HF received PC during fatal hospitalization (4% vs. 32% in cancer). Additionally, HF patients received PC later during their hospitalization (at the sixth day vs. third day in cancer) and closer to death: the median time during which HF patients received PC was four days compared to seven days in cancer. Numerous studies have shown that specialized palliative care is underutilized in the HF population, even in the dying phase. A study that used data from the National Health Service in Great Britain found that only 7% of patients with HF received PC before dying, compared to 48% of patients with cancer, and that those with HF received it much closer to death than patients with cancer [11]. Similarly, a study conducted in the United States found that patients with HF enroll in hospices at lower rates compared with patients with cancer and closer to death, typically around 3 days before death [12]. Underutilization can be attributed to several factors, including difficulties identifying the end-of-life phase in patients with HF, which has been commonly described as a barrier to delivering PC or end-of-life care to these patients [13, 14]. Interestingly, our data showed a longer LOS for patients who received PC across all diagnosis groups. This suggests that a less steep decline with a longer LOS facilitates the identification of a terminal phase, initiation of comfort measures and treatment, and referral to PC. In fact, we found that the odds of being prescribed opioids increased nine times for each hospitalization day. Although the most marked association between LOS and medication was for opioids, a longer LOS was also positively and statistically significantly associated with being prescribed the remaining three types of medication. Not only does it take time to identify the active dying phase, but a PC intervention also takes time to be effective and bring significant benefits [15, 16]. If patients with HF tend to experience steep clinical decline, it is of great importance to provide early PC in this specific population.

Another factor that could have contributed to the low PC referral among “HF” patients is the lack of awareness and training among cardiologists regarding the potential benefits of PC in patients with HF [17–20]. Addressing these barriers by educating and training all HF professionals in basic PC skills may be crucial to improving the integration of PC in the management of this disease, especially during the last days of life.

Finally, this study shows only moderate medical integration between cardiology and specialized palliative care services in our institution. This is often the case in other institutions and countries. The latest European atlas of PC services showed that only eight countries on the continent have cardiology services offering PC and that collaboration between both services rarely occurs [21]. Moreover, a study carried out in Italy showed not only a few services offering specialized palliative care, but end-of-life care of any kind [22]. Our data show the need to strengthen cooperation between cardiology and specialized palliative care services and to promote a PC approach (generalist PC) in the practice of cardiologists, internists, general practitioners, nurses, and other health care professionals.

### Sex and gender differences in specialized palliative care referrals

Although approximately half of the patients with HF included in our study were women, only 25% of those who received PC were women. That is, men with HF received three times as much PC as women with HF. This finding is interesting, as several studies have shown that women with HF have more symptoms, more unmet needs, and a lower quality of life than men with HF [23, 24]. In addition, women often outlive their male partners, which has been described as one of the causes why men die mainly at home in the care of their wife and family or in the hospital if there is a situation that the caregiver does not know how to handle at home. Women, in contrast, are often more institutionalized than men and therefore die mostly in such institutions [25]. In fact, a study conducted in Switzerland showed that in the German region of the country in which our hospital is located, most women die in nursing homes irrespective of age and have half the odds of dying in a hospital as compared to men [26]. Nursing homes or similar institutions usually do not have PC specialists. Therefore, to ensure equitable access to specialized palliative care, it is important to implement mobile palliative care services.

### Strengths and limitations

We used administrative records to identify all patients who had died in a tertiary hospital from cancer or HF for seven years. Therefore, even after applying the exclusion and inclusion criteria, we managed to include data from almost 3,000 patients in our analysis. This significant sample size allowed us to compare the medications not only by disease or palliative care status, as is traditionally done, but we could also test the differences across the interactions between both characteristics (disease category and specialized palliative care status). This type of analysis allowed us to identify the biggest determinants of medication prescriptions.

Unfortunately, we did not have information about the treating specialty. For example, many of the patients with HF at our institution are hospitalized in the internal medicine ward. This could also be the case for patients with cancer. Therefore, we cannot draw conclusions about the prescription practice in oncology or cardiology but only draw conclusions according to the diagnosis and whether or not they received specialized palliative care.

Finally, we did not include HF-related variables, such as cardiac function measures, functional class, or symptoms, in our regression analysis for further adjustment.

## Conclusion

Across the diagnostic groups, a notable difference in the usage of the four medication types was observed between the PC + and PC- patients. This difference was more pronounced among patients with HF (without cancer). These results suggest that the prescription of these medications is mainly in the hands of palliative care specialists, and on a non-palliative care specialist level, these medications are incorporated more into the routine care of patients with cancer than in patients with heart failure. This study underscores the need to provide early specialized palliative care to patients with heart failure due to their shorter hospitalization, which allows little room to adequately identify and treat the end of life.

## Electronic supplementary material

Below is the link to the electronic supplementary material.

Supplementary Material 1

## Data Availability

Anonymized data will be available upon reasonable request.
