# Supplementary material for: Prescription of essential medication during the final hospitalization of patients with heart failure or cancer
Source: BMC Palliat Care. 2025 Feb 26;24:51. doi: 10.1186/s12904-025-01682-w (PMC11863666; doi:10.1186/s12904-025-01682-w)
Supplement: Supplementary file 1 — Supplementary Material 1 [file 12904_2025_1682_MOESM1_ESM.docx]

# Supplementary material

Contents

[Supplementary material 1. ICD-10 codes used to create categorical disease variables 2](#_Toc171334680)

[Heart failure 2](#_Toc171334681)

[Cancer 2](#_Toc171334682)

[Chronic kidney disease 2](#_Toc171334683)

[Chronic obstructive pulmonary disease 2](#_Toc171334684)

[Dementia 2](#_Toc171334685)

[Supplementary material 2. Medications included within each medication category 3](#_Toc171334686)

[Opioids 3](#_Toc171334687)

[Benzodiazepines 3](#_Toc171334688)

[Antipsychotics 3](#_Toc171334689)

[Anticholinergics 3](#_Toc171334690)

## Supplementary material 1. ICD-10 codes used to create categorical disease variables

### Heart failure

I09.81, I11.0, I11.01, I13.00, I13.20, I50.0, I50.00, I50.01, I50.02, I50.03, I50.04, I50.05, I50.1, I50.11, I50.12, I50.13, I50.14, I50.19, I50.2, I50.20, I50.21, I50.22, I50.23, I50.3, I50.30, I50.31, I50.32, I50.33, I50.4, I50.40, I50.41, I50.42, I50.43, I50.8, I50.81, I50.810, I50.811, I50.812, I50.813, I50.814, I50.82, I50.83, I50.84, I50.89, I50.9

### Cancer

C90.00, C31.0, D47.4, C50.8, C02.1, C15.4, C83.3, C34.3, C71.5, C04.0, C16.3, C67.9, C61, C81.2, C25.0, C24.0, C83.7, C79.5, C34.0, C78.6, C50.9, C67.2, C83.1, C22.1, C57.0, C92.00, C91.00, C38.4, C15.5, C80.0, D46.9, C34.1, C77.8, C44.4, C25.9, C85.2, C51.9, C44.2, C78.7, C18.1, C79.3, C06.1, C92.50, C84.7, C48.0, C67.3, C16.0, C71.1, D01.5, C25.2, C67.8, C34.2, D18.02, C22.0, C34.8, C37, C18.0, C19, C85.1, C78.0, C17.0, C17.2, C18.7, C75.1, C34.9, C64, C56, C93.00, C55, C92.10, C20, C73, C16.5, C25.1, C43.5, C50.4, C40.2, C18.2, C70.0, C83.8, C81.7, C24.1, C71.8, C53.8, C84.4, C90.20, C16.2, C47.3, C18.4, C51.8, C92.80, C71.2, C06.2, C49.1, C49.5, C86.5, C49.4, C18.6, C11.8, C94.00, C90.10, C43.7, C43.9, C92.30, C49.2, C77.2, C92.90, C54.1, C44.7, C13.9, C01, C53.9, C45.0, C40.0, C03.1, C81.9, C23, C78.2, C18.9, C80.9, C76.2, C68.0, C95.00, C09.9, C12, C71.3, C18.3, C06.8, C67.4, C32.1, C41.2, C02.9, D46.7, C84.0, C32.8, C16.1, C15.9, C86.3, C49.8, C68.9, C44.3, C25.7, C43.6, C79.7, C49.0, C02.3, C60.1, C45.1, C62.1, C91.10, C09.0, C82.0, C66, C16.9, C15.8, C93.10, C49.6, C91.50, C25.8, C86.2, C32.0, C04.9, C24.9, C76.1, C21.8, C78.4, C86.0, C10.8, C11.9, C22.3, C79.84, C68.8, C04.1, C17.9, D46.5, C41.4, C71.0, C81.1, C03.0, C79.83, C48.1, C47.4, C26.9, C78.8, C07, C21.1, C43.4, D46.2, C86.6, C65, C92.01, C49.3, C47.9, C54.9, C71.6, C83.0, C50.5, C92.40, C26.0, C02.0, C11.1, C92.51, C47.1, C25.3, C18.5, C02.4, C05.0, C16.4, C13.2

### Chronic kidney disease

N18, N18.1, N18.2, N18.3, N18.30, N18.31, N18.32, N18.4, N18.5, N18.6, N18.9

### Chronic obstructive pulmonary disease

J44, J44.0, J44.00, J44.01, J44.02, J44.03, J44.09, J44.10, J44.11, J44.1, J44.8, J44.80, J44.9, J44.91, J44.99

### Dementia

F00.0, F00.1, F00.2, F00.9, F01.8, F01.9, F02.0, F02.1, F02.3, F02.8, F03, F05.1, G30.8, G30.9, G30.0, G30.1, G31.0, G31.1

## Supplementary material 2. Medications included within each medication category

### Opioids

Morphine, Apomorphine, Buprenorphine, Butorphanol, Codeine, Dihydrocodein, Diamorphine, Fentanyl, Bupivacain/Fentanyl, Ropivacain/Fentanyl, Hydrocodone, Hydromorphone, Levomethadone, Meperidine, Methadone, Normethadone, Oxycodone, Oxycodone naloxon, Oxymorphone, Pentazocine, Remifenatil, Tapentadol, Tramadol, Tramadol+paracetamol

### Benzodiazepines

Midazolam, Alprazolam, Bromazepam, Clonazepam, Diazepam, Flunitrazepam, Flurazepam, Lorazepam, Lormetazepam, Nitrazepam, Oxazepam, Triazolam

### Antipsychotics

Haloperidol, Chlorpromazine, Levomepromazine, Olanzapine, Quetiapine, Risperidone, Ziprasidone

### Anticholinergics

Glycopyrronium bromide, Scopolamine butylbromide (hyoscine), Atropine, Scopolamine transdermal patch
